# 177. User Preferences for Visualization of Antibiogram Data in Clinical Practice for Empiric Prescription of Antibiotics

**DOI:** 10.1093/ofid/ofab466.379

**Published:** 2021-12-04

**Authors:** Alexandria R Vingino, Peter Rabinowitz, Hema Kapoor, Vickie Ramirez, Ann Salm

**Affiliations:** 1 University of Washington - Center for One Health Research, Seattle, Washington; 2 University of Washington, Seattle, WA; 3 Quest Diagnostics, Secaucus, New Jersey; 4 Quest Diagnostics, Incorporated, Secaucus, New Jersey

## Abstract

**Background:**

Antibiograms are widely used to present antibiotic susceptibility data, but user preferences for data visualization have received little attention. We report on a qualitative research study designed to gauge preferences for presenting antibiotic resistance data, with the goals of improving speed and effectiveness of prescribing empiric antibiotics in out-patient practices to meaningfully influence antibiotic stewardship programs.

**Methods:**

Criteria for online focus groups included having the ability to prescribe antibiotics, practice in Washington state, and familiarity with antibiogram usage. A preliminary survey (Fig. 1) was sent to selected participants to understand their role in healthcare and their current attitudes towards antibiograms. During focus groups, we presented examples of 3 antibiograms: standard (Fig. 2A), color-coded for % susceptible (Fig. 2B), and color-coded for change in % susceptible from 2013 to 2016 (Fig. 2C).

Figure 1. Preliminary Survey via RedCap

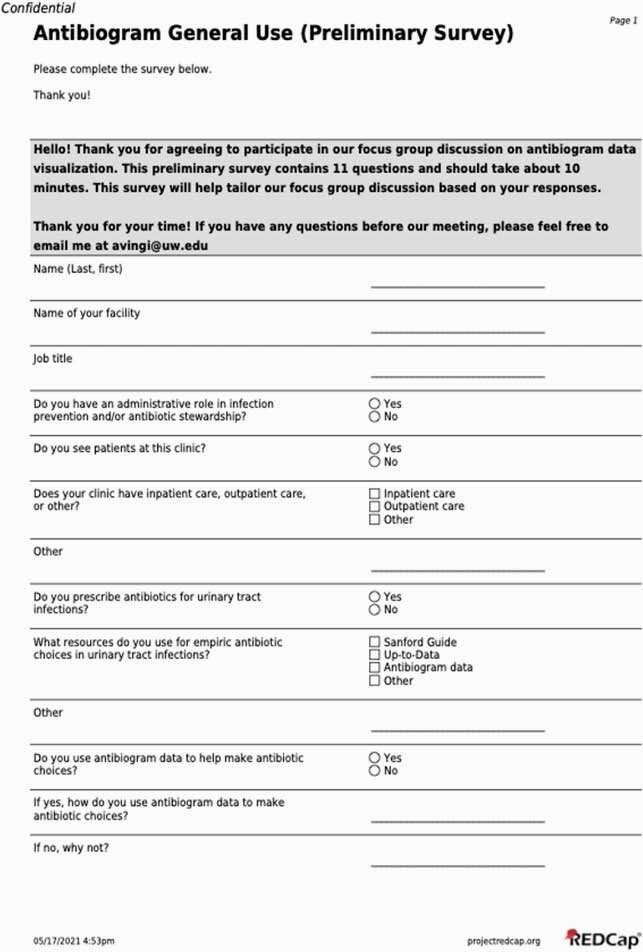

A preliminary survey via RedCap was sent all focus group participants to capture current attitudes towards antibiograms and antibiotic resistance data.

Figure 2. Presented antibiograms for focus group discussions using Quest Data. (A) Standard antibiogram for displaying % susceptibility. (B) Antibiogram color-coded for % susceptibility. (C) Antibiogram color-coded for change in % susceptibility, comparing 2013 data to 2016 data.

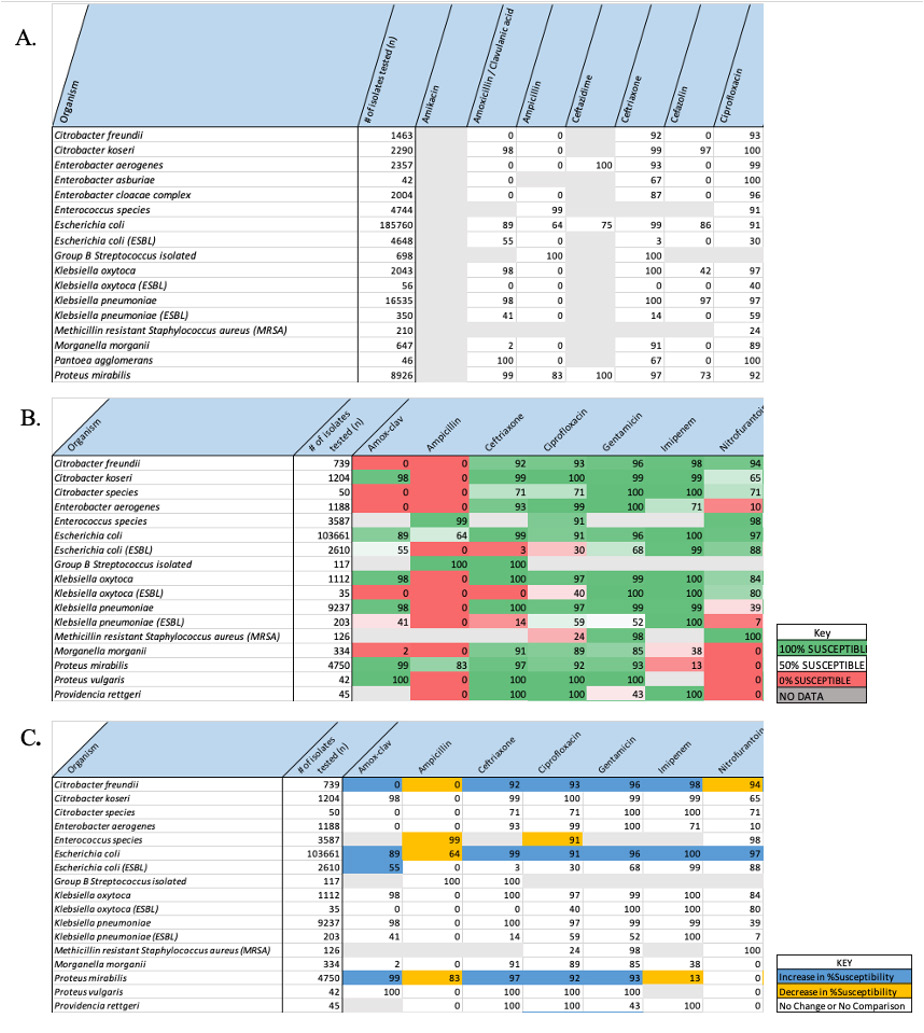

**Results:**

Focus groups were held between October 2020 and March 2021. Participants were 44 years of age on average, with 6-23 years of experience in primary care and/or infectious disease practice. Eight of nine participants took the preliminary survey. The survey revealed that 5 (63%) participants used antibiograms in their practice. Most participants (7; 88%) preferred an online format to print out antibiogram tables. Discourse analysis from focus groups (n=3) revealed common themes regarding Figures 2A-C as examples of antibiograms. Key ideas included discussion of the data source and content, arrangement of the table, usability during clinical days, and efforts for antibiotic stewardship related to antibiogram use. All focus group participants (n=9) favored the feature of color-coding cells and found the data in the Fig. 2B user friendly. Consensus across all groups was that antibiogram tables would not be useful for daily practice. Clinicians would rather receive simplified therapy suggestions either in the patient laboratory report or in the electronic health system.

**Conclusion:**

Antibiograms can be useful for visualization of empirical data but can become a more useful tool if they can be interpreted and simplified for guiding empiric prescribing in daily out-patient practice.

**Disclosures:**

**Hema Kapoor, MD; D(ABMM**), **Quest Diagnostics** (Employee, I am an employee of Quest Diagnostics and receive its stock as part of my employment.) **Ann Salm, M (ASCP), MSc, PhD**, **Quest Diagnostics** (Employee, I am an employee of Quest Diagnostics and receive its stock as part of my employment.)

